# Thyroxine restores severely impaired cutaneous re-epithelialisation and angiogenesis in a novel preclinical assay for studying human skin wound healing under “pathological” conditions ex vivo

**DOI:** 10.1007/s00403-020-02092-z

**Published:** 2020-06-22

**Authors:** H. Post, J. E. Hundt, G. Zhang, R. Depping, C. Rose, E. A. Langan, R. Paus

**Affiliations:** 1 Department of Dermatology, University of Münster, Münster, Germany; 2 Department of Dermatology, University of Lübeck, Lübeck, Germany; 3 Lübeck Institute for Experimental Dermatology, Lübeck, Germany; 4 Department of Plastic and Reconstructive Surgery, Shanghai Ninth People’s Hospital, Shanghai, China; 5 Institute of Physiology, University of Lübeck, Lübeck, Germany; 6 Centre for Dermatology Research, University of Manchester and NIHR Manchester Biomedical Research Centre, Manchester, UK; 7 Dr. Phillip Frost Department of Dermatology and Cutaneous Surgery, University of Miami Miller School of Medicine, Miami, FL USA; 8 Monasterium Laboratories, Münster, Germany

**Keywords:** Wound healing, Hypoxia, Skin model, Ex vivo

## Abstract

Impaired cutaneous wound healing remains a major healthcare challenge. The enormity of this challenge is compounded by the lack of preclinical human skin wound healing models that recapitulate selected key factors underlying impaired healing, namely hypoxia/poor tissue perfusion, oxidative damage, defective innervation, and hyperglycaemia. Since organ-cultured human skin already represents a denervated and impaired perfusion state, we sought to further mimic “pathological” wound healing conditions by culturing experimentally wounded, healthy full-thickness frontotemporal skin from three healthy female subjects for three days in either serum-free supplemented Williams’ E medium or in unsupplemented medium under “pathological” conditions (i.e. hypoxia [5% O_2_], oxidative damage [10 mM H_2_O_2_], absence of insulin, excess glucose). Under these “pathological” conditions, dermal–epidermal split formation and dyskeratosis were prominent in organ-cultured human skin, and epidermal reepithelialisation was significantly impaired (*p* < 0.001), associated with reduced keratinocyte proliferation (*p* < 0.001), cytokeratin 6 expression (*p* < 0.001) and increased apoptosis (*p* < 0.001). Moreover, markers of intracutaneous angiogenesis (CD31 immunoreactivity and the number of of CD31 positive cells and CD31 positive vessel lumina) were significantly reduced. Since we had previously shown that thyroxine promotes wound healing in healthy human skin ex vivo, we tested whether this in principle also occurs under “pathological” wound healing conditions. Indeed, thyroxine administration sufficed to rescue re-epithelialisation (*p* < 0.001) and promoted both epidermal keratinocyte proliferation (*p* < 0.01) and angiogenesis in terms of CD31 immunoreactivity and CD31 positive cells under “pathological” conditions (*p* < 0.001) ex vivo. This demonstrates the utility of this pragmatic short-term ex vivo model, which recapitulates some key parameters of impaired human skin wound healing, for the preclinical identification of promising wound healing promoters.

**Electronic supplementary material:**

The online version of this article (10.1007/s00403-020-02092-z) contains supplementary material, which is available to authorized users.

## Introduction

Chronic skin ulceration, particularly in the context of an ageing population, is a complex challenge in daily medical practice and is associated with (i) significant morbidity, (ii) impaired quality of life and (iii) substantial health care costs [1]. Indeed, in Germany alone, the estimated annual costs associated with the treatment of leg ulceration reach almost 9000 Euros (= 9900 USD) per patient. With approximately 330,000 patients being treated annually, the result is an annual cost of approximately 3 billion Euros (3.3 billion USD) [25, 26]. Leg ulcer management costs in the U.S. are considerably higher and are expected to increase further, not least due to the epidemic-like increase in diabetic foot ulcers [27].

Effective wound healing (WH) is a metabolically demanding process that relies upon the co-ordinated action of multiple tightly regulated complex biological processes, such as the timely initiation and termination of inflammation, adequate cell proliferation and efficient tissue remodelling, all of which are dependent upon sufficient tissue oxygenation, energy supply and innervation [17, 18, 22, 62]. Dysregulation of the WH process, at any level, may result in failure to re-establish tissue integrity, culminating in the development of a chronic wound [17, 18]. Moreover, co-morbidities such as diabetes mellitus, obesity and peripheral vascular disease not only contribute to the development of chronic wounds but may also hinder their clinical resolution [38].

Despite the presence of a multitude of both in vivo [20, 61, 69] and in vitro [48, 49, 59, 67] WH models, the development of optimally suited preclinical WH research models that not only sufficiently reflect in vivo WH conditions in patients with multiple chronic co-morbidities but which can also predict WH-promoting agents remains a key challenge in the field of cutaneous WH research [45]. Instead, research efforts have focussed on the development of sophisticated murine WH models, including diabetic mice models [42], in which the effects of specific diseases on WH can be investigated. However, given the recognised differences between cutaneous WH in mice and humans, the clinical relevance of these models to human WH is, at best, limited.

Furthermore, the available human WH models typically lack most resident skin cell populations and skin appendages, for example, the hair follicle (HF) [2, 16] and, therefore, fail to represent full “skin equivalents.” Experimentally wounded human skin organ culture models [41, 43, 73] contain all of key resident cutaneous cell populations, native extracellular matrix whose WH-facilitating functions are very challenging to reconstitute [50] and skin appendages as well as a structurally intact, though no longer perfused, skin vasculature. Therefore, human skin organ culture systems are not only closest to the clinical in vivo situation but also constitutively recapitulate two important aspects of impaired WH [30, 33, 75] in that they are non-perfused and functionally denervated [74]. Using serum-free culture conditions [37, 41, 75], these ex vivo WH models can be easily standardised, optimising reproducibility, and circumventing the confounding influences of various serum-dependent WH-modulatory factors [75]. Furthermore, these models can be utilised to investigate the aspects of angiogenesis, central to effective WH [34, 74].

However, even these models often fail to recapitulate the factors complicating WH in vivo, namely hypoxia, oxidative stress and hyperglycaemia. Therefore, based on previously published full-thickness human skin WH ex vivo models [31, 34, 37, 41, 74, 75], we sought to further simulate “pathological” WH conditions typically found under healing-impaired conditions, namely hyperglycaemia, hypoinsulinaemia, hypoxia and oxidative stress, that may be found in diabetic leg ulcers, by applying this to short-term organ-cultured human skin. Given the evidence that the HF may accelerate WH and that it may be hair cycle dependent [2, 28, 39], we specifically sought to establish and validate our model using skin rich in terminal HFs, namely frontotemporal scalp skin.

In the second part of our study, we sought to ascertain the extent to which an agent known to promote WH in the human full-thickness skin ex vivo WH model, namely thyroxine (T4), also exerts WH-promoting effects in the “pathological” WH model. For this, we chose to test T4 as the lead compound, because it has been shown to promote cutaneous WH both in mice in vivo [53, 54] and in healthy human skin ex vivo [74], and has an extensively characterised toxicological profile in humans [32]. Indeed, this proof-of-principle part of the study was designed to determine whether the model could preclinically identify candidate WH-promoting agents, which exerted their effects despite conditions known to profoundly impair WH.

## Materials and methods

### Organ culture of experimentally wounded human skin under “standard” and “pathological” healing conditions

Temporal and occipital region scalp skin samples from six females (aged 52–65 years) were obtained as by-products from cosmetic surgical procedures. All patients provided informed consent, and the study was approved by the Institutional Research Ethics Committee at the University of Luebeck (University of Luebeck, license: Reference 06-109). All experiments were performed in accordance with the ethical standards set by the Declaration of Helsinki and its later amendments.

Skin samples from the first three patients were used to establish a “pathological” WH model and skin samples from the remaining subjects were used to test the effects of T4, under standard and “pathological” conditions (see below), on several WH-related parameters. Skin fragments were “wounded” via a 4-mm punch biopsy, with the tissue periphery, which rapidly shows signs of epiboly, i.e. the tendency of the epithelium to rapidly enclose traumatically exposed mesenchyme, [10, 58] taken as the “wound edge”. This approach was in contrast to the established punch-in-a-punch design used to examine skin wounding ex vivo [41, 43] and was necessary given the extensive dermal–epidermal separation which occurred under “pathological” conditions.

The punch biopsies were then placed in standard supplemented Williams’ E medium (WE) (22), or in the modified “pathological” medium. All punches were cultured in a six-well plate, each well containing 3 ml of medium and two skin punches, floating at the liquid–air interface. After completion of the organ culture, skin punches were snap frozen in cryomatrix and liquid nitrogen and stored at − 80 °C until cryosections were cut, stained and analysed. See Supplementary Fig. 1 for the experimental setup.

**Fig. 1:**
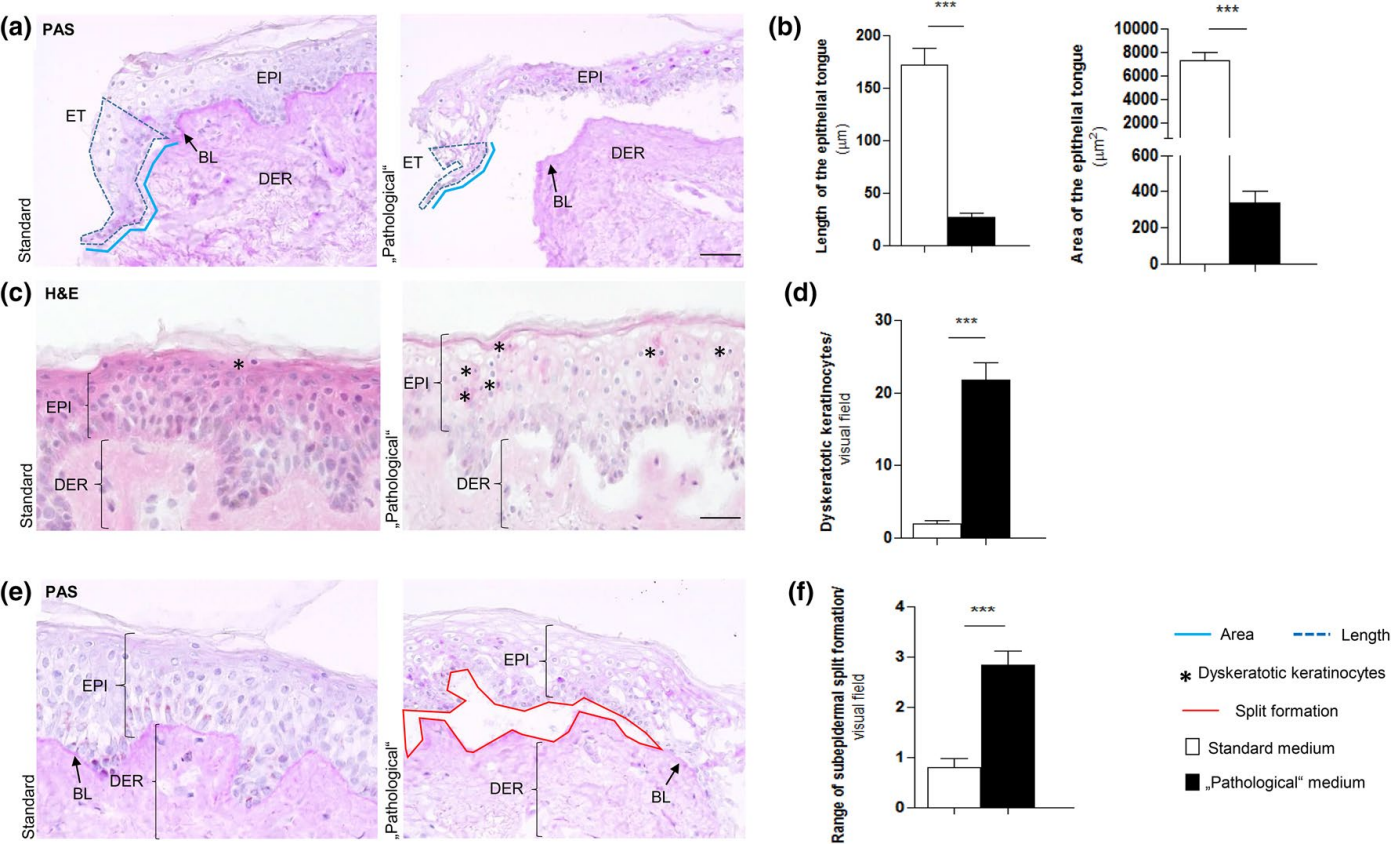
“Pathological” culture conditions resulted in major changes in epithelial morphology and severe reduction of epithelial tongue formation. **a** Periodic acid–Schiff (PAS) staining of left wound edges with a physiologically developed epithelial tongue (ET) under standard conditions and the smaller ET under “pathological” conditions. Evaluations were performed by measuring the marking of the length (light blue continuous line) and the area (dark blue dashed line). **b** Comparing the sizes (length and area) of the ETs from standard and “pathological” conditions revealed dramatic differences under “pathological” conditions. ***p ≤ 0.001; pooled data from three different patients, six punches, 26–29 skin sections; mean ± SEM; *p *value was calculated by Mann–Whitney U test for unpaired samples. **c** Haematoxylin and Eosin staining of skin punches from standard and “pathological” culture conditions showing the epidermis and labelled dyskeratotic keratinocytes (KCs) (black stars). **d** Dyskeratotic KCs were counted inside the whole epidermis per visual field. Dyskeratosis was a predominant phenomenon under “pathological” culture conditions. ***p ≤ 0.001; pooled data from three different patients, 5–6 punches, 23–44 skin sections; mean ± SEM; *p* value was calculated by Mann–Whitney *U* test for unpaired samples. **e** PAS staining represents the different manifestations of split formation (red continuous line) comparing standard and “pathological” cultured skin. **f** The extent of split formation was assessed by a semi-quantitative method per visual field, and it was significantly higher detectable in skin of “pathological” culture conditions. ***p ≤ 0.001; pooled data from three different patients, six punches, 38–44 skin sections; mean ± SEM; *p* value was calculated by Mann–Whitney *U* test for unpaired samples. *ET* epithelial tongue, *EPI*: epidermis, *DER*: dermis, *BL*: basal lamina. Scale bars: 50 μm

### “Standard” versus “pathological” skin organ culture medium

Serum-free WE supplemented with l-glutamine (2 mmol/l), hydrocortisone (10 ng/ml), insulin (10 μg/ml) and streptomycin (10 µg/ml)/penicillin G (100 IU/ml) served as the standard control culture medium [8, 37, 41]. Hydrocortisone is a necessary supplement of serum-free medium for ex vivo skin organ culture. The supplemented WE medium allows human skin to be cultured for up to two weeks [37].

The “pathological” WH conditions included hyperglycaemia, achieved by the addition of glucose. Given that diabetes can be diagnosed with a random serum glucose concentration of ≥ 11.1 mmol/dl (≥ 200 mg/dl), and that tissue damage due to hyperglycaemia is a long-term complication [4, 55], we selected a glucose concentration ten times of that used to diagnose diabetes to ensure an adequate effect in our short-term model. Given the pharmacological concentrations of glucose used to severely impair WH in short-term organ culture (which rather imitate hyperglycemic diabetic coma conditions than classical diabetes mellitus-associated chronic skin damage associated with peripheral neuropathy and diabetic microangiopathy), it is important to point out that we did not seek to fully simulate diabetic conditions ex vivo.

Hyperglycaemia results in the formation of advanced glycation end-products (AGEs). The formation of AGEs is also promoted by oxidative stress and leads to tissue dysfunction by the cross-linking of collagens, activation of macrophages and covalent bonding of lipoproteins [57]. Under “standard-diabetic” conditions of latent hyperglycaemia, the formation of AGEs takes several weeks [4] and were, therefore, unsuitable for our model. Profound over-supply of substrates can accelerate this process to a duration of only a few hours [55]. Accumulation of AGEs is one of the main pathomechanisms causing tissue damage in diabetic patients. Additionally, matrix glycosylation was identified as an underlying mechanism of an increased cellular apoptosis, inhibition of cell proliferation, migration and angiogenesis in the skin of diabetic patients [15]. Therefore, we applied a concentration of 138.8 mM (2500 mg/dl) of glucose to create hyperglycaemic circumstances. Importantly, this very high glucose serum concentration is pathologically relevant as it can be reached in patients undergoing hyperglycaemic coma. In addition, insulin was omitted to imitate hypoinsulinemic diabetic-like conditions.

Oxidative damage was experimentally induced by adding 3.06 μl of hydrogen peroxide (H_2_O_2_) per well (concentration 10 mM) based on our prior experience with experimental induction of oxidative damage in human skin appendages [24, 72]. Finally, hypoxia was generated by incubation under 5% oxygen, using a hypoxia incubator (Ruskinn InVivo400) [9] instead of the standard oxygen supply of 20.9% in ambient air.

To determine whether the “pathological” model is capable of identifying candidate agents that promote human skin WH even under severely impaired WH conditions ex vivo, we tested T4 as the lead compound since this peptide hormone promotes skin WH, both in mice in vivo [53, 54] and in healthy human skin ex vivo [74].

Test groups were supplemented with T4 at a concentration of 100 nM to promote mitochondrial activity and to reduce standard ageing-associated read out parameters in human skin ex vivo [65]; this T4 concentration also promotes mitochondrial activity and biogenesis in human scalp HF [66]. Moreover, we had previously shown that 100 nM T4 enhances hair matrix keratinocyte (KC) proliferation and prolongs anagen hair growth [64], both of which are expected to exert overall WH-promoting effects [2, 39]. Given that the very limited availability of human skin for organ culture precluded running a dose–response study, 100 nM T4 appeared to be an optimally chosen test concentration. This concentration could also be easily reached after topical application under clinical conditions.

### Staining methods and quantitative (immuno-)histomorphometry

Phenotypic changes in the subcorneal epidermis were quantified by counting the total number of cells demonstrating the morphological features of dyskeratosis, i.e. premature keratinisation with a pyknotic, hyperchromatic nucleus and lightened cytoplasm (“halo”) [46]. Dyskeratotic cells were recognized by routine haematoxylin and eosin (H&E) histochemistry. The extent of subepidermal split formation was evaluated by determining the degree of separation along the entire epidermis. We assessed re-epithelialisation by quantifying the length and area of the neoepidermis [“epithelial tongue” (ET)] on day 3 as previously described [31, 34, 74]. Quantitative histomorphometry for split formation and re-epithelialisation was performed after histochemical Periodic acid–Schiff (PAS) staining to facilitate demarcation of the basal lamina (BL) zone, also in the newly formed neoepidermis [41]. For each parameter, we selected pre-defined areas for the evaluations as follows: dyskeratosis and the dermal–epidermal split formation were measured along the skin punch. The length and area of the epithelial tongue was measured in two visual fields (both edges) and the number of Ki-67/TUNEL-positive cells inside the epithelial tongues was measured in addition to the number of Ki-67/TUNEL-positive cells in three additional visual fields. Cytokeratin 6 (CK6) immunoreactivity (IR) was measured in the entire epidermis skin punch and the angiogenesis parameters were measured in the dermis adjacent to the ETs and in one visual field in the middle of the skin punch.

Evaluation was performed with Image J software by measuring the length and area of the ET. Photomicrographs were obtained using the Keyence-Biozero 8000. Dimensions were orientated via the insertion of a 50 µm scale bar. Immunofluorescence (IF) microscopy for the employed read-out parameters (Ki-67/ TUNEL, CK6 and CD31) was performed and analysed as described previously [37, 41].

Proliferation and apoptosis were determined by Ki-67/TUNEL double-IF staining. The total number of Ki-67, TUNEL and DAPI was counted in a defined area inside the epidermis and ETs. In the first experimental set-up, Ki-67 positive and TUNEL positive  cells were counted inside a defined area of the ET but also in three additionally defined areas of epidermis. In the second experimental set-up (i.e. addition of T4), evaluation of apoptosis and proliferation was restricted to the ETs.

CK6 expression was measured given that it is increased in both hyperproliferative and wounded epithelium [52, 70] and given that a thyroid hormone (TH) response element is located in the promoter region of the CK6 gene [71].

Angiogenesis, another key WH parameter, was assessed by the detection of CD31 positive cells, a validated endothelial cell marker [40, 74]. Three angiogenesis parameters were analysed, namely (i) CD31 immunoreactivity (IR), (ii) the total number of CD31positive nuclei and (iii) the total number of CD31 positive lumina. All three criteria were analysed in two predefined dermal visual fields directly underneath the neoepithelial tongue. CK6 and CD31 IR were normalised to the control group [41, 74]. The antibodies used, and their concentrations, are available in supplementary Tables 1 and 2. All pictures were analysed with Image J software (National Institutes of Health, Bethesda, MD).

### Statistical analyses

All data sets were tested for normal distribution using Graph Pad Prism v. 3.00 (Graph-Pad Software Chicago, IL, USA). When the data set was normally distributed, the *t*-test was used when comparing two groups. When the data were not normally distributed, the Mann–Whitney *U* test was selected. Statistical analyses were also performed using Graph Pad Prism v. 3.00 (Graph-Pad Software Chicago, IL, USA). All data were expressed as mean ± SEM (standard error of the mean); *p* values * < 0.05, ** ≤ 0.01, *** ≤ 0.001 were regarded significant. For each parameter, two punches were cultured and compared. All data were pooled, resulting in a total of 19–54 skin fragments per experimental group, derived from three different female patients to establish the model and from three female patients to test the effect of thyroxine. Data pooling was justified since the obtained results in at least three independently run experiments showed comparable trends.

## Results

### “Pathological” wound healing culture conditions induce characteristic morphological changes including dyskeratosis, dermal–epidermal separation and impaired re-epithelialisation

First, we tested the working hypothesis that “pathological” WH conditions would negatively effect skin structure and integrity as well as re-epithelialisation, even in short-term organ culture. After wounding, KCs migrate from the BL along the wound edge to restore the epithelial barrier after wounding, [18] which can be assessed qualitatively and quantitatively by measuring the formation of ETs, i.e. neoepidermis formation [41, 74]. Indeed, “pathological” WH conditions almost completely prevented this process (Fig. 1a, b). Importantly, however, at least some residual reepithelialisation capacity was preserved.

Moreover, the number of intraepidermal dyskeratotic cells and the extent of the epidermal separation from the BL(epidermolysis) were both significantly increased in the skin under “pathological” WH conditions compared to standard human skin organ culture conditions (Fig. 1c–f). This likely reflected severe tissue damage resulting from metabolic, osmotic and oxidative stress since increased cell size, nuclear shifting and vacuolation of KCs during tissue hypoxia have been previously described under these conditions [60].

### “Pathological” wound healing conditions induce apoptosis and inhibit proliferation and cytokeratin 6 expression of epidermal keratinocytes ex vivo

**Fig. 2:**
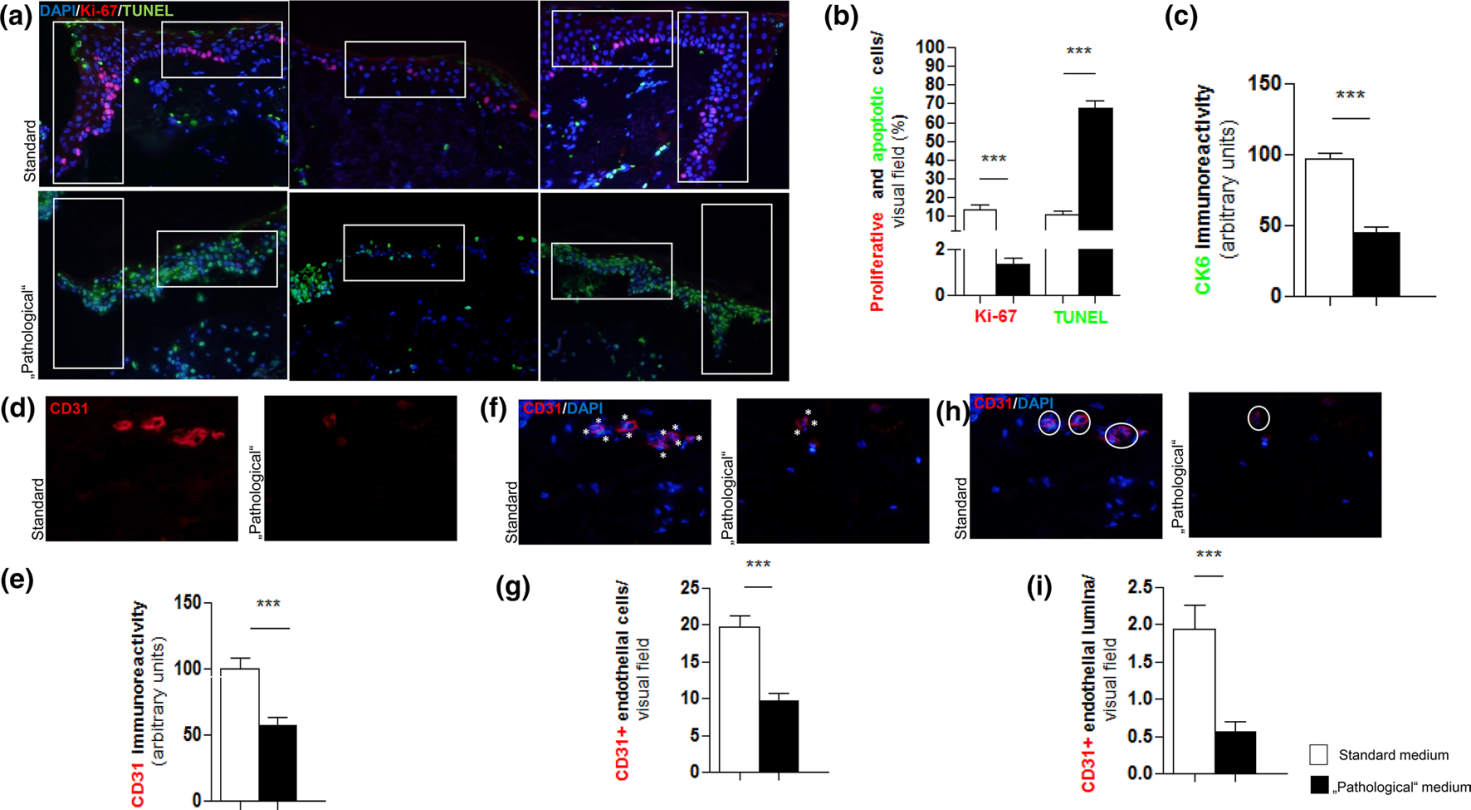
The effect of “pathological” medium conditions on keratinocyte proliferation, cytokeratin 6 and neoangiogenesis. **a** Double immunofluorescent Ki-67/TUNEL/DAPI staining of left and right epithelial tongues (ETs) and the epidermis. Red fluorescent immunoreactivity (IR) represents Ki-67 positive (proliferating), green fluorescent IR represents TUNEL positive (apoptotic) and blue fluorescent IR represents DAPI positive (all nuclei) which were counted separately in defined reference areas (white boxes). **b** The amount of proliferative and apoptotic cells is provided as percentages of all cells (number of DAPI-positive cells was set as 100%) inside the reference area from the ETs and epidermis. The number of proliferative cells is significantly lower and apoptosis is highly increased under “pathological” conditions. ***p ≤ 0.001; pooled data from three different patients, six punches, 31–35 skin sections; mean ± SEM; *p* value was calculated by Mann–Whitney *U* test for unpaired samples. **c** Cytokeratin 6 (CK6) protein expression in the “pathological” cultured skin was significantly reduced compared to normalised results of the standard medium (= 100%). ***p ≤ 0.001; pooled data from three different patients, six punches, 34–35 skin sections; mean ± SEM; *p* value was calculated by Mann–Whitney *U* test for unpaired samples. **d **and** e** Immunofluorescent CD31 staining of an analysed part of the dermis demonstrating a clear red fluorescent CD31-positive signal whilst this is hardly detectable in the skin of “pathological” medium conditions. The immunoreactivity (IR) was assessed per visual field of three defined dermal reference areas comparing “pathological” medium conditions to normalised results of the standard medium conditions (= 100%). CD31 IR was significantly less detected in the “pathological” sections. **f** and **g** An endothelial cell was counted for each blue fluorescent DAPI-positive nucleus associated with a red fluorescent CD31-positive signal (white stars) which were sparsely found under “pathological” medium conditions. The number of endothelial cells was assessed per visual field of three defined dermal reference areas comparing the results of “pathological” medium conditions to the results of standard medium conditions. The number of endothelial cells was significantly lower under “pathological” conditions. Immunofluorescent CD31/DAPI staining of a defined dermal area. An endothelial lumen (white encircled) was counted for blue-fluorescent DAPI-positive nuclei associated with a red-fluorescent CD31-positive signal forming a lumen. The number of lumina was assessed per visual field of three defined dermal reference areas comparing the results of “pathological” medium conditions to the results of standard medium conditions. **h** and **i** The number of lumina was significantly lower under “pathological” conditions. Scale bar: 50 μm; ***p ≤ 0.001; pooled data from three different patients, six punches, 41–42 skin sections; mean ± SEM; *p* value was calculated by Mann–Whitney *U* test for unpaired samples

Next, we examined how “pathological” WH conditions influenced epidermal KC apoptosis and proliferation ex vivo. KC apoptosis was significantly increased throughout the entire epidermis under “pathological” conditions compared to that in control conditions (Fig. 2a, b). Moreover, the “pathological” conditions resulted in decreased KC proliferation, in line with evidence of severely reduced cell viability and the disruption of epidermal morphology and epithelial migration shown in Fig. 1. However, substantial residual proliferative KC activity was still present even under these conditions, attesting to sustained epidermal viability after 3 days of organ culture even under these severely impaired WH conditions ex vivo.

Given that CK6 protein expression is strongly upregulated in wounded human epidermis and during epidermal regeneration [41] and that this keratin actively impacts KC migration during wound healing [52], we also tested whether “pathological” medium conditions also affect CK6 expression. Indeed, CK6 IR, measured in the ET and epidermis, was decreased under “pathological” WH conditions (Fig. 2c). This further attests to the WH-impaired status of experimentally wounded human epidermis under these assay conditions.

### Angiogenesis is impaired under “pathological” wound healing conditions

To complete the characterisation of our “pathological” WH model, we asked whether hyperglycaemia, hypoinsulinaemia, hypoxia and oxidative stress also impact upon angiogenesis since we had had previously shown that indications of angiogenesis are still visible, albeit a fluctuating levels, even after several days of organ-culturing human skin under serum-free conditions [34, 74]. Markers of angiogenesis were significantly downregulated under “pathological” medium conditions, as measured by assessing (i) total CD31 immunoreactivity, (ii) the number of CD31 + endothelial cells, and (iii) the number of CD31 positive blood vessel cross sections (i.e. with a discernible vessel lumen) per visual field (Fig. 2d-i).

In summary, these findings demonstrate that the simple, easily set up organ culture system for experimentally wounded skin reported here rapidly generates severely healing-impaired previously healthy human skin within just three days ex vivo. Dyskeratosis and split formation were measured to confirm that the skin was being sufficiently stressed by the “pathological” conditions. Having confirmed this, we next addressed the ability of T4 to influence the key parameters of WH, namely re-epithelialisation and neo-angiogenesis under “pathological” conditions.

### Thyroxine promotes the proliferation and Cytokeratin 6 expression of human epidermal keratinocytes, stimulates angiogenesis, and markedly reduces keratinocyte apoptosis ex vivo

Most WH assays employed in vitro, ex vivo, or in vivo examine the impact of test agents and experimental manipulations under WH conditions that reflect acute WH in essentially healthy tissue. Unsurprisingly, WH-promoting effects seen under these more or less physiological conditions often do not translate into tangible clinical benefits under the only WH conditions that really matter in clinical practice: impaired WH and ulcer formation [45]. Therefore, the litmus test for any candidate WH-promoting agent or treatment strategy is that it unfolds significant WH benefits, such as the acceleration and improvement of epidermal repair and WH-associated angiogenesis, also under clinically relevant WH-impaired conditions.

We, therefore, explored whether or not the candidate WH-promoting thyroid hormone, T4 [54, 74], positively impacts epidermal repair and angiogenesis and sought to examine the effect of T4 during “pathological” conditions, given its documented WH-promoting effect, albeit ex vivo [19, 29, 54].

**Fig. 3:**
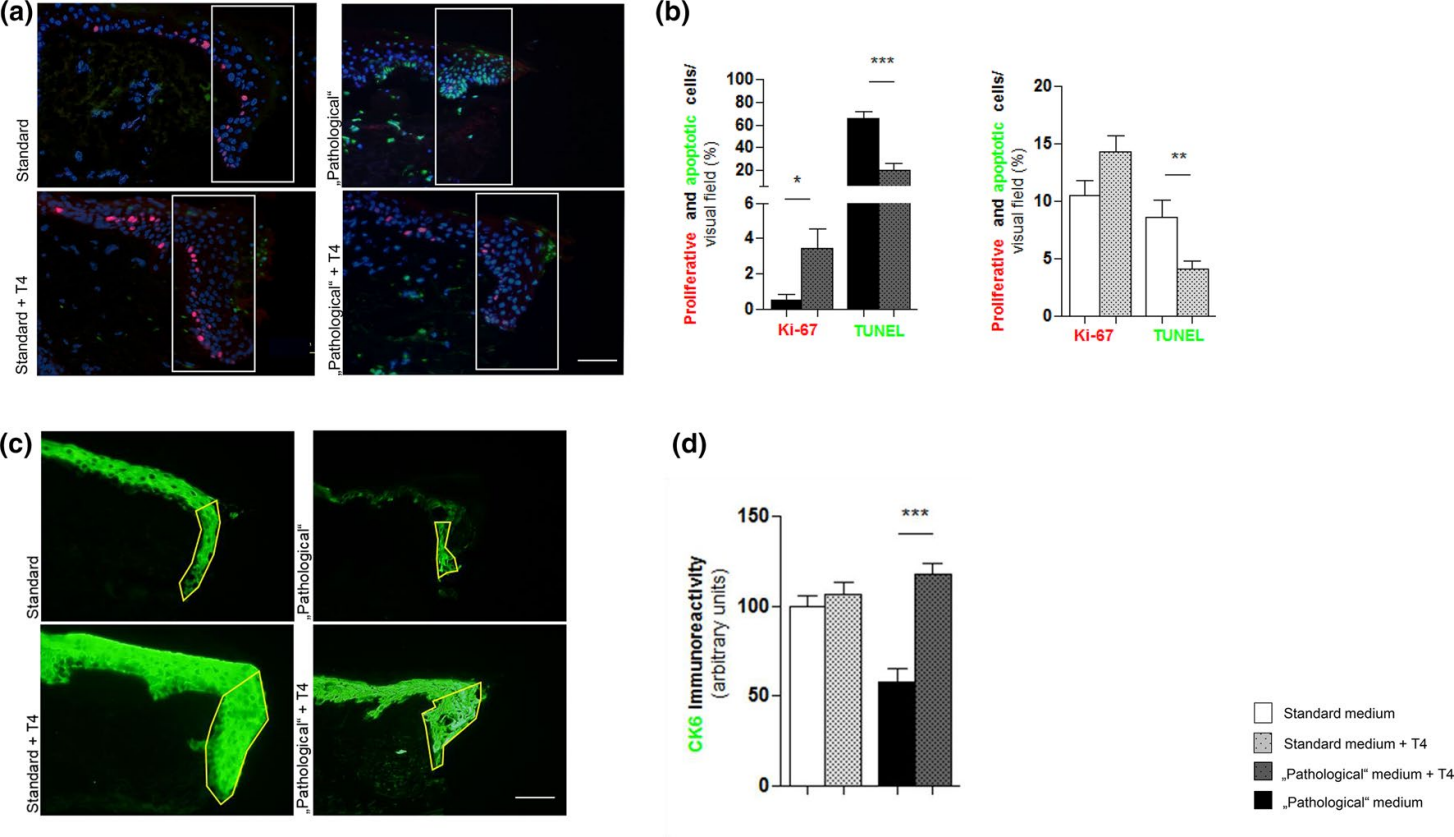
Keratinocyte proliferation is increased by thyroxine (T4) and cytokeratin 6 (CK6) protein expression was reduced under “pathological” conditions and upregulated when T4 was added to the medium. **a** Double-immunofluorescent Ki-67/TUNEL/DAPI staining showing right wound edges and epidermal tongues (ETs). Statistical analyses of the amount of proliferative/apoptotic cells comparing standard versus (vs.) standard + T4 conditions and “pathological” vs. “pathological” + T4 conditions. Results of proliferative and apoptotic cells are provided as percentage of all cells (number of DAPI-positive cells was set as 100%) inside the reference area from the ETs. **b** The amount of proliferative cells tended to be increased under standard + T4 medium conditions but was only significant inside the ETs of “pathological” + T4 medium conditions. Apoptosis was reduced under T4-treated medium conditions most significantly under “pathological” medium conditions. ***p ≤ 0.001, **p ≤ 0.01, *p < 0.05; pooled data from three different patients, six punches, 24–35 skin sections; mean ± SEM; *p* value was calculated by Mann–Whitney *U* test for unpaired samples. Scale bar: 50 μm. **c** Immunofluorescent CK6 staining of skin sections showing right wound edges and ETs. Green fluorescent immunoreactivity (IR) represents the CK6-positive signal and appears brighter in the skin from T4-supplemented medium conditions. The CK6-positive IR was assessed in the ETs (yellow continuous line) by quantitative immunofluorescence morphometry. **d** Statistical analyses demonstrate the assessment of the CK6 IR comparing standard versus (vs.) standard + T4 conditions and “pathological” vs. “pathological” + T4 conditions. All results were normalised to the standard medium (= 100%). Upregulation of CK6 IR was significant under “pathological” conditions. ***p ≤ 0.001; pooled data from three different patients, six punches, 34–35 skin sections; mean ± SEM; *p* value was calculated by Mann–Whitney *U* test for unpaired samples. Scale bar: 50 μm

Interestingly, the addition of 100 nM T4 for 3 days to the medium dramatically reduced epidermal KC apoptosis under standard (*p* < 0.01) and under “pathological” (0.001) conditions (Fig. 3) and also significantly increased KC proliferation under “pathological” conditions (*p* < 0.05) culture conditions ex vivo. Moreover, the inhibitory effects of these culture conditions on ET formation (reflecting epidermal KC migration and epidermal repair) were reversed by T4 supplementation (Supp. 1). In addition, T4 tended to restore the reduced CK6 expression in the ET under “pathological” WH conditions (Fig. 3c, d).

**Fig. 4:**
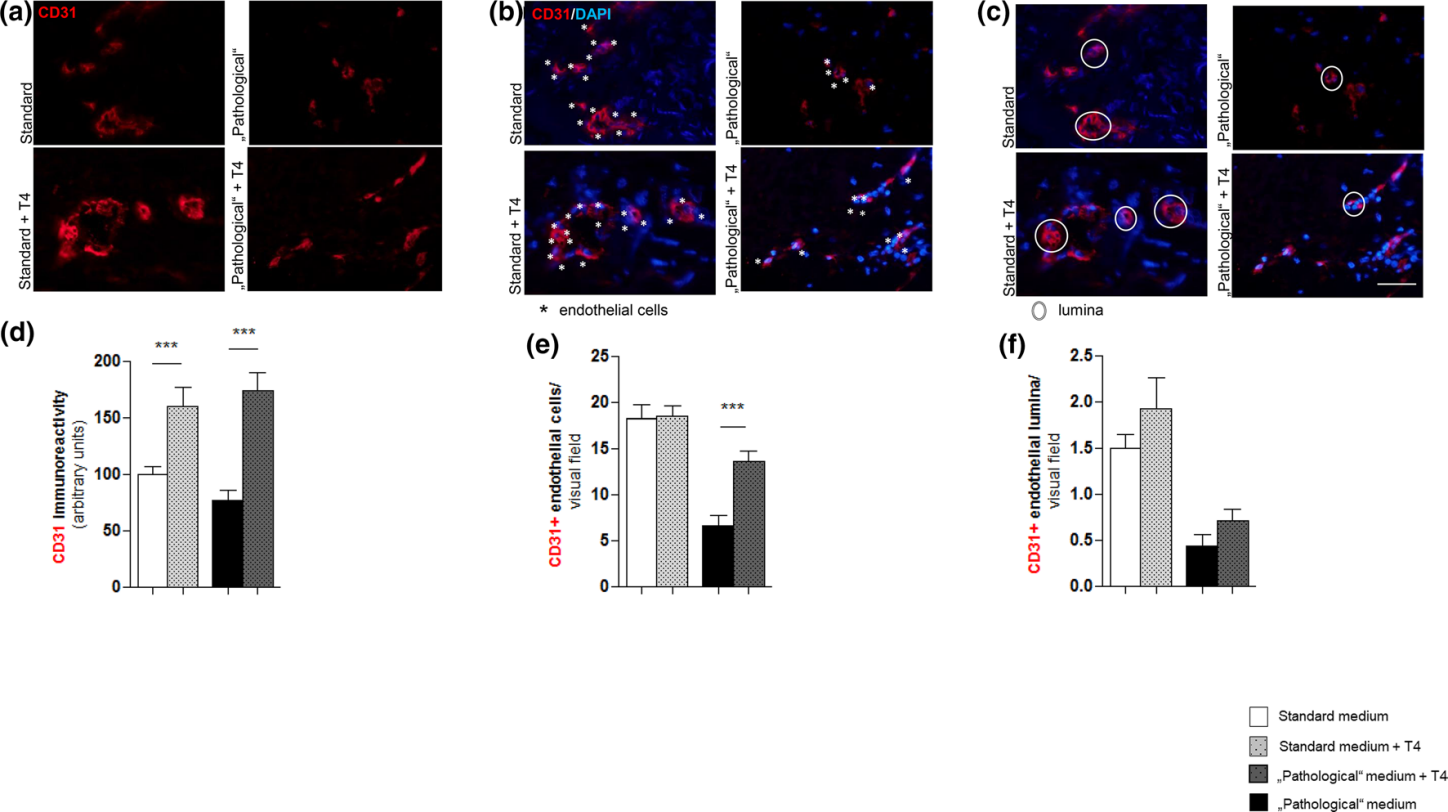
Intracutaneous angiogenesis is strongly impaired but may be partially ameliorated by thyroxine (T4) under “pathological” conditions. **a**, **b** Immunofluorescent CD31 staining of an analysed part of the dermis demonstrating a brighter red-fluorescent CD31-positive signal in the skin of T4-supplemented medium conditions. Comparing the immunoreactivity (IR) of standard versus (vs.) standard + T4 conditions and “pathological” vs. “pathological” + T4 conditions normalised to the mean value of standard conditions to 100%. The T4-supplemented groups show a significant upregulation of the CD31 positive IR which is even more obvious in “pathological” conditions. **c**, **d** Immunofluorescent CD31/DAPI staining of a defined dermal area. An endothelial cell was counted for each blue-fluorescent DAPI-positive nucleus associated with a red-fluorescent CD31-positive signal (white stars). The number of endothelial cells was assessed per visual field of three defined dermal reference areas comparing the results of standard vs. standard + T4 conditions and “pathological” vs. “pathological” + T4 conditions. The number of CD31-positive endothelial cells was not significantly different comparing standard and standard + T4 but in the T4-supplemented “pathological” group, significantly higher than in the “pathological” untreated group. **e**, **f** Immunofluorescent CD31/DAPI staining of a defined dermal area. An endothelial lumen (white encircled) was counted for blue-fluorescent DAPI-positive nuclei associated to a red-fluorescent CD31-positive signal forming a lumen. There was no significant difference of CD31-positive lumina, neither in the standard vs. standard + T4 nor in the “pathological” vs. “pathological” + T4 even though the T4-supplemented groups tended to contain a higher amount of lumina. Scale bar: 50 μm; ***p < 0.001; pooled data from three different patients, 39–54 skin sections; mean ± SEM; Mann–Whitney *U* test for unpaired samples

T4 addition to the medium resulted in both significantly increased CD31 IR and enhanced the number of CD31 positive cells under “pathological” conditions” (Fig. 2d–i). However, T4 did not significantly affect the number of blood vessel cross sections even though there was a statistically non-significant increase **(**Fig. 4a–f). Thus**,** while T4 appears to have restored some of the negative impact of the tested experimental “pathological” WH conditions on intracutaneous angiogenesis ex vivo, it remains unclear whether angiogenesis was really promoted.

## Discussion

The development of novel WH-promoting treatments is hampered by the lack of clinically relevant WH models in general, and the lack of WH models replicating WH in patients with multiple co-morbidities (diabetes, peripheral vascular disease) in particular. Therefore, we sought to establish a robust and clinically relevant “pathological” WH model and determine the extent to which candidate WH-promoting agents could modulate WH under these carefully defined and reproducible conditions.

For example, hyperglycaemia and hypoinsulinaemia or insulin end-organ resistance are known to impair WH via a reduction in dermal fibroblast proliferation and migration. Furthermore, the low-grade inflammation and angiogenesis associated with diabetes and the metabolic syndrome, respectively, also contribute to impaired WH and tissue remodelling [21]. In addition, the chronic tissue hypoxia and the accumulation of H_2_O_2_ and reactive oxygen species (ROS), resulting from reduced perfusion in peripheral vascular disease, also disrupts WH in vivo [56].

Here we have developed and validated a pragmatic, well-defined, serum-free human skin organ culture model, in which the epidermal repair and angiogenesis can be studied under severely healing impaired (“pathological”) WH conditions that mimic ex vivo, at least to some extent, the hypoxia, oxidative damage, impaired perfusion and innervation, hyperglycaemia and hypoinsulinaemia which may be present in diabetic ulcers in vivo. This model recreates impaired WH-associated alterations in KC morphology, reduced KC proliferation and migration, increased KC apoptosis, epidermolysis, and reduced angiogenesis and is, therefore, well suited for the preclinical testing of candidate WH-promoting agents that hope to positively affect these parameters. That T4 was able to partially “rescue” epidermal repair and promote angiogenesis, even under these severely healing-impaired clinically relevant WH conditions, in a human model, is interesting and potentially clinically relevant.

With the aim of validating our “pathological” WH model of human skin, we selected T4 as a candidate WH promotor based on its effects on mitochondrial biogenesis, KC proliferation and angiogenesis [36, 68]. The thyroid gland-derived hormone T4 is converted in peripheral tissues to the active hormone T3 and regulates metabolism. In terms of the skin, TH receptor (TH-R) is expressed in the human HF [64]; TH-dependent signalling regulates the expression of selected keratins, can prolong the anagen phase of hair growth and stimulate hair matrix KC proliferation [47, 64]. It has also been reported that thyroid stimulating hormone (TSH) treatment promotes the proliferation of human epidermal KCs and dermal fibroblast [14]. Epidermal barrier function is maintained via upregulation of dermal TH-R [3]. Furthermore, TH reportedly stimulates angiogenesis in vivo [74]*.*

The pro-angiogenic effect of T4 is mediated by the cell surface receptor integrin αvβ3. Stimulation of this receptor on endothelial cells stimulates intracellular transcription of pro-angiogenic growth factors (GFs), like bFGF and VEGF [13].

Induced hypothyroidism in mice results in papillary rarefaction and could be treated via systemically applied tri-iodothyronine (T3) which induced sprouting angiogenesis in the heart tissue of the hypothyroid mice [12]. There is also evidence that TH can initialise angiogenesis in vitro using the “HUVEC”-model under serum-free medium conditions, supplemented by T4 100 nM [36].

Specifically, in terms of WH, T4 has been shown to have a WH-promoting effect compared to untreated groups in animal studies [19, 54]. T3 enhanced WH processes in guinea pigs most likely by wound contraction [29]. T4 may represent an intriguing candidate WH promoter [7] especially if topical application can be delivered without affecting circulating T4 concentrations [11]. Additional potential WH-promoting agents, including interleukin 22 [6], pro-insulin C-peptide [35], and erythropoietin [5, 23] could also be preclinically tested in this clinically relevant WH model.

In terms of dissecting the molecular mechanism underlying the effect of T4 on WH in this model, future studies may wish to examine the extent to which fibroblast and HF stem cell proliferation contribute to the WH evidence in the model, for example, by determining matrix metalloproteinase, heat-shock protein and CK 15 expression, respectively. Indeed, therapies aimed at mobilising stem cells have recently be shown to promote cutaneous WH in diabetic rats [51]. Moreover, THs have been shown to not only regulate expression, apoptosis, and differentiation in human HF epithelial stem cells in situ and in vitro [63] but thyroid analogues also prolong the anagen phase of the hair cycle; a process intimately associated with WH [2, 44].

In summary, we report the development of a pragmatic organ culture assay that quickly transforms viable biopsy-wounded human skin into a “pathological” state, where WH is severely impaired. We have attempted to provide proof-of-principle that (i) human skin with severely “pathological” WH features is still capable of responding in a therapeutically desired manner to treatment with a candidate WH promoter and (ii) TH-R agonists are interesting, but as yet insufficiently investigated candidate promoters of “pathological” WH. This novel preclinical test system, which is characterised by severe hypoxia, excessive ROS levels, hyperglycaemia and insulin withdrawal, can serve as a surrogate assay for probing the efficiency of candidate WH promoters under well-standardized and clinically relevant ex vivo conditions.

## Electronic supplementary material

Below is the link to the electronic supplementary material.

Supplementary file1 **Fig**. **1** The demonstration of the experimental set-up. Full thickness human frontotemporal skin fragments were cultured in different medium conditions of clinically physiological (= standard) and “pathological” conditions treated or untreated by thyroxine (T4). (A) The 4 mm punch biopsy obtained from skin fragments after cosmetic surgery. Ki-67 stained images show the location of analysed areas inside the punch. (B) The composition of the “pathological” medium, Williams’ E. medium serves as a basic medium and was modified to induce the specific culture condition. (C) The timetable of the experimental setup. **Fig**. **2** Human organ cultured skin influenced by “pathological” factors reveal major differences in epidermal/dermal structure and T4 likely has a positive influence on keratinocyte migration. (A) Split formation (SF) (B) Dyskeratosis (C) length and (D) Area of the epithelial tongues (ETs). SF analysed by Periodic acid-Schiff staining was categorised into 4 stadiums: (Stage 4) > ¾ of the epidermis is detached, (Stage 3) > ½ of the epidermis is detached, (Stage 2) < ½ of the epidermis is detached and (Stage 1) < ¼ th of the epidermis is detached. Data of patient 2 and 3 show a very similar degree of SF in the “pathological” medium (mean-p.m., patient 2: 3.9 mean-p.m., patient 3:3.85). Patient 1 also showed a higher but non-significant difference between standard and “pathological” medium. Dyskeratosis was concise under “pathological” culture conditions. Haematoxylin and Eosin stained sections of standard and “pathological” conditions were evaluated. Measurement of the ETs by Image J were standardised (scale bar: 50 µm). The length and area of the ET varied dependent on the culture medium and whether T4 was added or not. Dyskeratosis, SF and ETs were analysed per visual field along the epidermis, detached and attached epidermis. Data are mean ±SEM of 3 patients. Significance relative to control data (standard medium) at the same time point denoted by *p<0.05 , **p<0.01, ***p<0.001, Mann-Whitney U test, t-test. **Fig**. **3** Primary and secondary antibodies. (PPTX 3475 kb)
