# Supplementary material for: Thyroxine restores severely impaired cutaneous re-epithelialisation and angiogenesis in a novel preclinical assay for studying human skin wound healing under “pathological” conditions ex vivo
Source: Arch Dermatol Res. 2020 Jun 22;313(3):181–92. doi: 10.1007/s00403-020-02092-z (PMC7935818; doi:10.1007/s00403-020-02092-z)
Supplement: Supplementary file 1 — Supplementary file1 Fig. 1 The demonstration of the experimental set-up. Full thickness human frontotemporal skin fragments were cultured in different medium conditions of clinically physiological (= standard) and “pathological” conditions treated or untreated by thyroxine (T4). (A) The 4 mm punch biopsy obtained from skin fragments after cosmetic surgery. Ki-67 stained images show the location of analysed areas inside the punch. (B) The composition of the “pathological” medium, Williams’ E. medium serves as a basic medium and was modified to induce the specific culture condition. (C) The timetable of the experimental setup. Fig. 2 Human organ cultured skin influenced by “pathological” factors reveal major differences in epidermal/dermal structure and T4 likely has a positive influence on keratinocyte migration. (A) Split formation (SF) (B) Dyskeratosis (C) length and (D) Area of the epithelial tongues (ETs). SF analysed by Periodic acid-Schiff staining was categorised into 4 stadiums: (Stage 4) > ¾ of the epidermis is detached, (Stage 3) > ½ of the epidermis is detached, (Stage 2) < ½ of the epidermis is detached and (Stage 1) < ¼ th of the epidermis is detached. Data of patient 2 and 3 show a very similar degree of SF in the “pathological” medium (mean-p.m., patient 2: 3.9 mean-p.m., patient 3:3.85). Patient 1 also showed a higher but non-significant difference between standard and “pathological” medium. Dyskeratosis was concise under “pathological” culture conditions. Haematoxylin and Eosin stained sections of standard and “pathological” conditions were evaluated. Measurement of the ETs by Image J were standardised (scale bar: 50 µm). The length and area of the ET varied dependent on the culture medium and whether T4 was added or not. Dyskeratosis, SF and ETs were analysed per visual field along the epidermis, detached and attached epidermis. Data are mean ±SEM of 3 patients. Significance relative to control data (standard medium) at the same time point denoted b [file 403_2020_2092_MOESM1_ESM.pptx]

## Slide 1
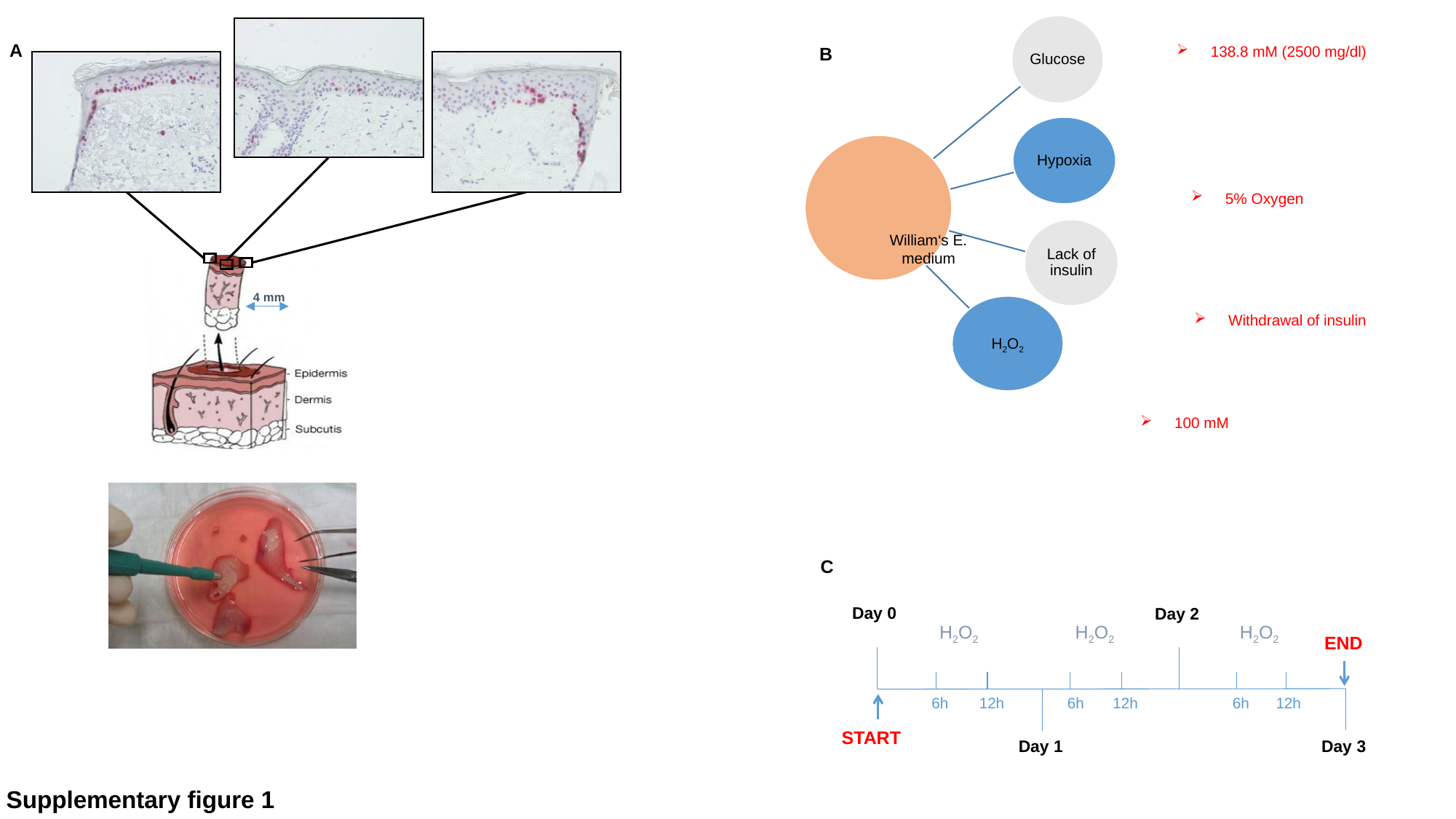

138.8 mM (2500 mg/dl)
5% Oxygen
William‘s E.
medium
Withdrawal of insulin
100 mM
4 mm
A
B
C
Day 0
Day 2
H2O2
H2O2
H2O2
END
6h
12h
6h
12h
6h
12h
START
Day 1
Day 3
Supplementary figure 1

## Slide 2
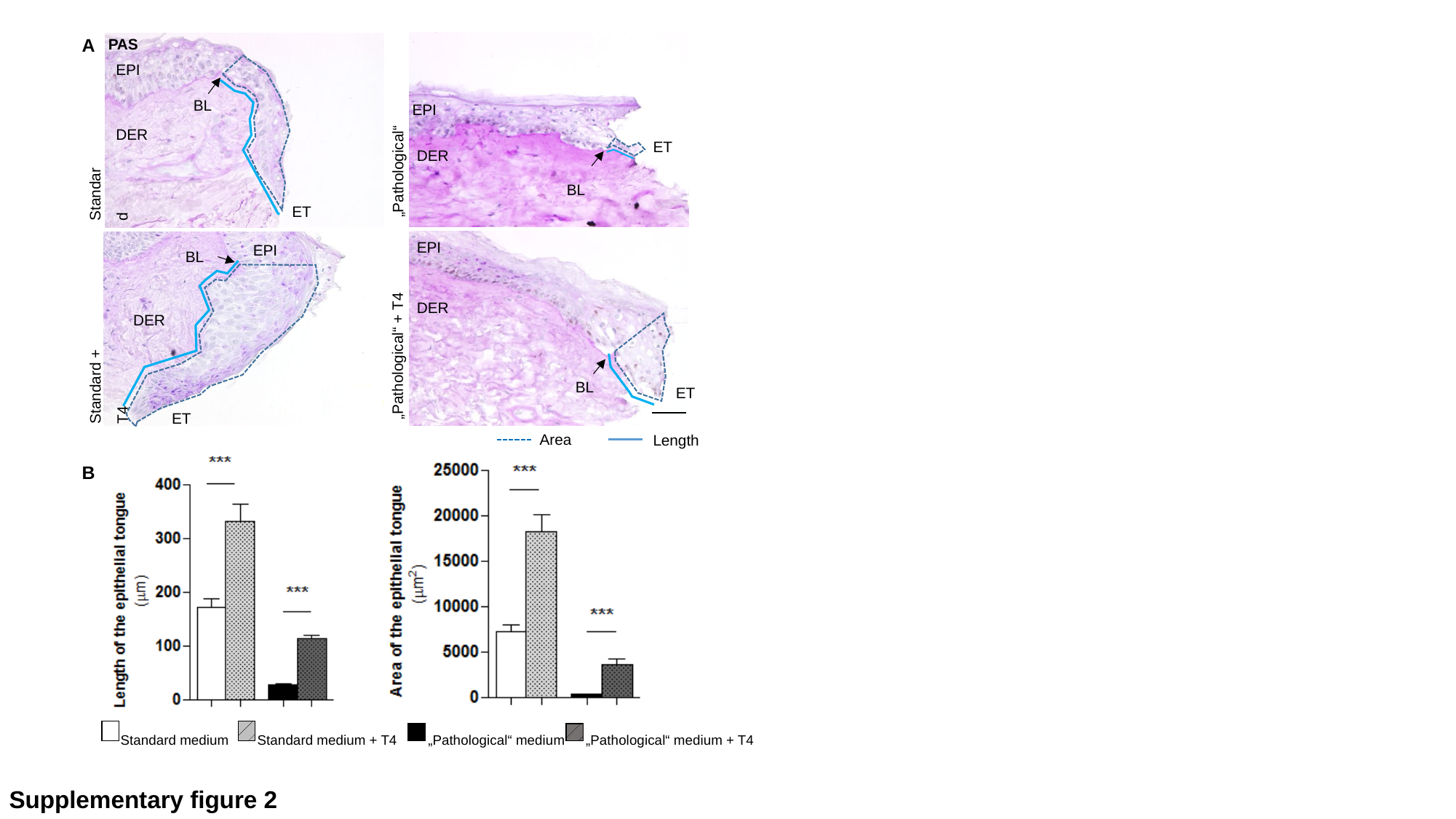

A
PAS
EPI
BL
EPI
„Pathological“
DER
ET
DER
Standard
BL
ET
EPI
EPI
BL
„Pathological“ + T4
DER
DER
Standard + T4
BL
ET
ET
Area
Length
B
Standard medium
Standard medium + T4
„Pathological“ medium
„Pathological“ medium + T4
Supplementary figure 2

## Slide 3
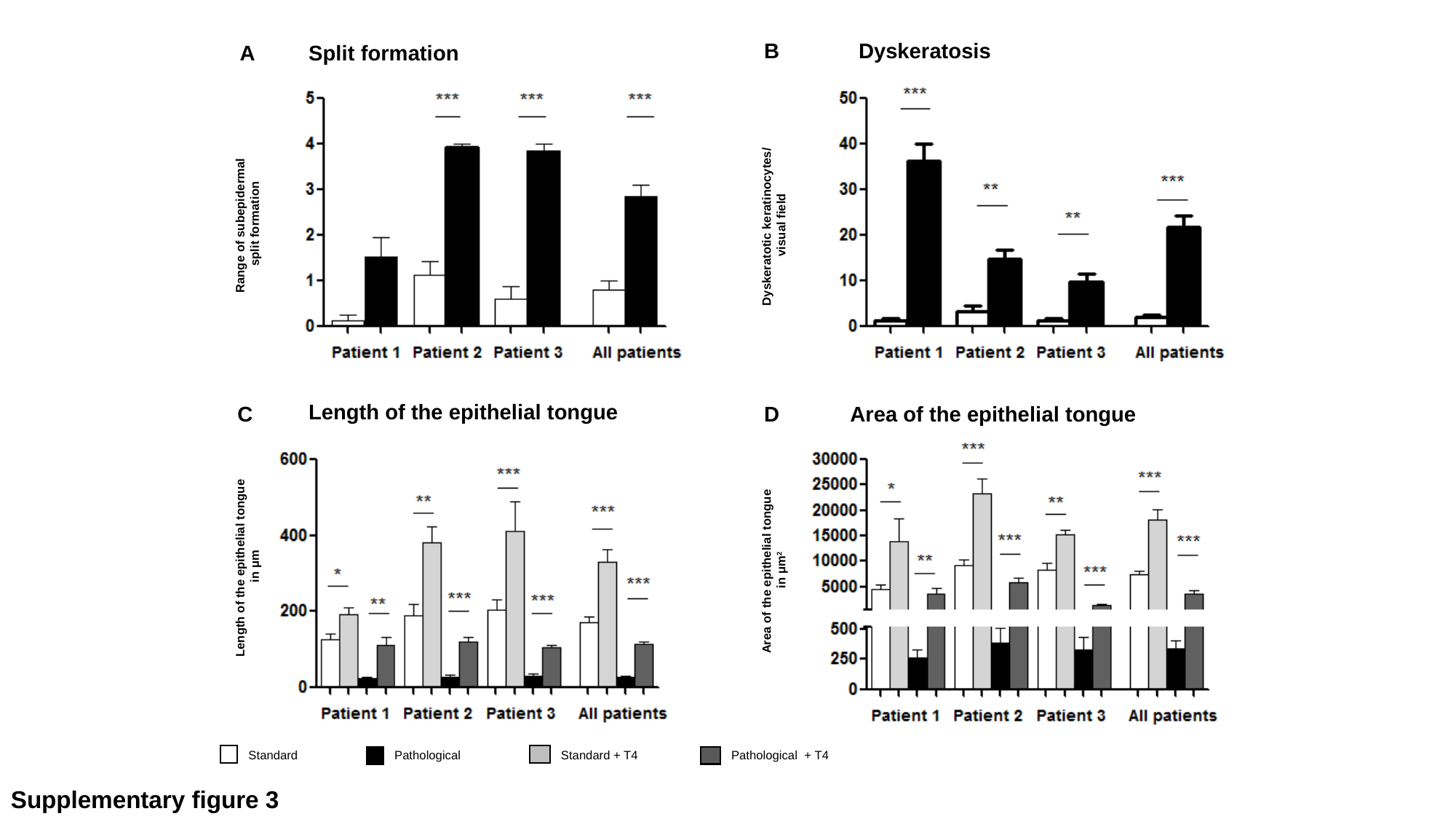

B
Dyskeratosis
A
Split formation
Range of subepidermal
 split formation
Dyskeratotic keratinocytes/
 visual field
Length of the epithelial tongue
C
D
Area of the epithelial tongue
Length of the epithelial tongue
in μm
Area of the epithelial tongue
in μm2
Pathological + T4
Standard + T4
Pathological
Standard
Supplementary figure 3

## Slide 4
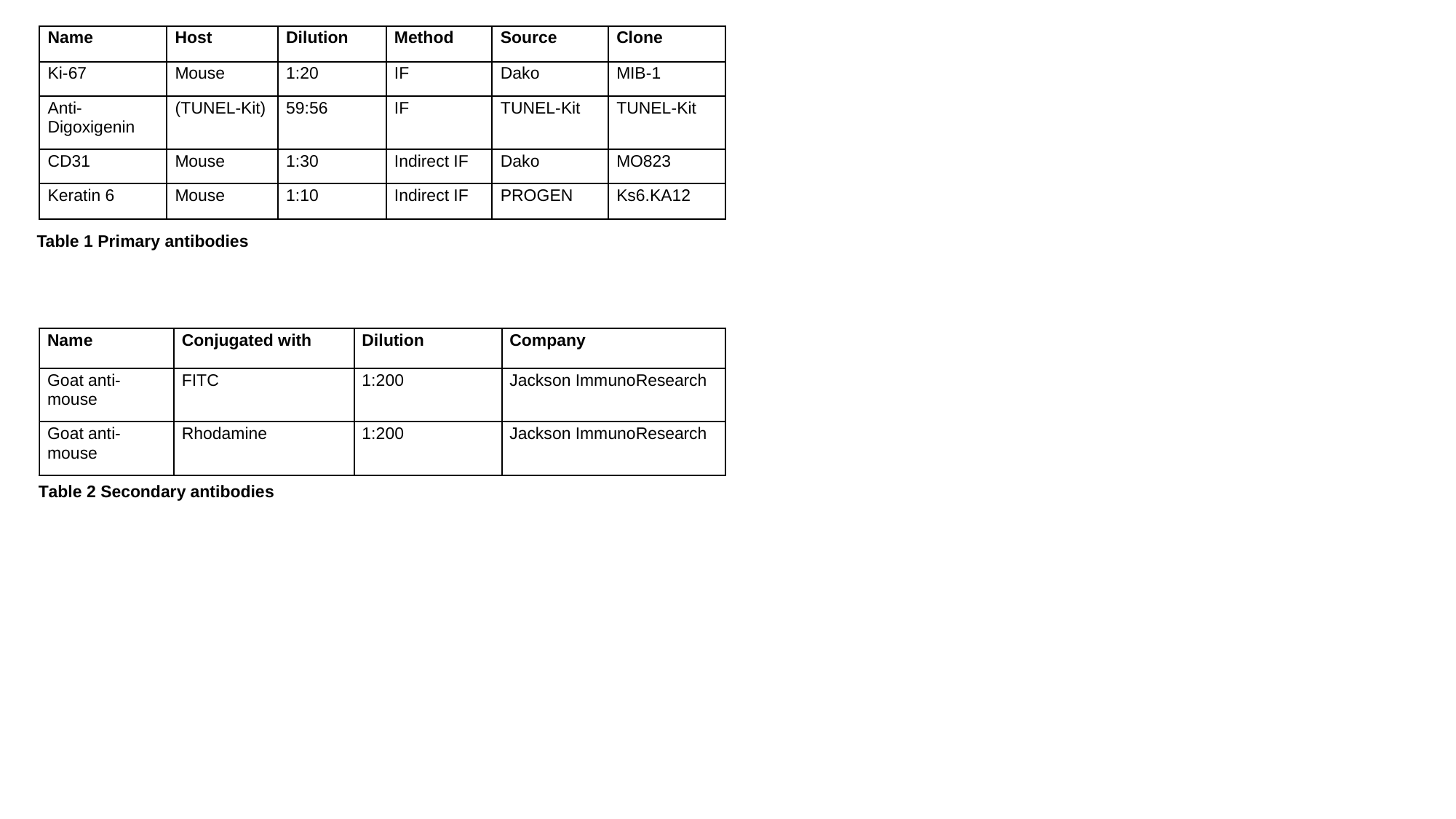

Table 1 Primary antibodies
